# A randomized controlled trial on the application of a chronic disease management platform based on digital health technology combined with an innovative model of intelligent management for hypertension in patients with hypertension

**DOI:** 10.3389/fdgth.2025.1678235

**Published:** 2026-01-26

**Authors:** Keli Liu, Benshan Niu, Xiaoyi Zhang, Lingyuan Zhang, Yuexia Gao, Juying Lu

**Affiliations:** 1 Department of Health Management, School of Public Health, Nantong University, Nantong, Jiangsu, China; 2 Joint Division of Clinical Epidemiology, Affiliated Hospital of Nantong University, School of Public Health of Nantong University, Nantong, China; 3 Health and Disease Management Center, Affiliated Hospital of Nantong University, Nantong, Jiangsu, China; 4 Department of Emergency Medicine, Affiliated Hospital of Nantong University, Nantong, China

**Keywords:** chronic disease management, digital health technology, health education, hypertension, patient self-management

## Abstract

**Importance:**

Digital health technology (DHT)-based chronic disease management platforms combined with smart hypertension models may improve patient self-management.

**Objective:**

To compare the effect of Nantong University Affiliated Hospital's DHT platform combined with an intelligent hypertension management model (providing education, follow-up, evaluation) vs. traditional offline management on patients' systolic blood pressure (SBP).

**Design, setting, and participants:**

This was a two-arm, parallel-group randomized controlled trial conducted from July 2023 to March 2025. Participants were adults (≥18 years) with hypertension and uncontrolled blood pressure.

**Interventions:**

Participants were randomly assigned using computer-generated sequences to an integrated digital health platform with intelligent hypertension management (intervention, *n* = 285) or to traditional offline management (control, *n* = 285).

**Main outcomes:**

Primary outcome: SBP at 12 months. Secondary outcomes: Diastolic blood pressure (DBP), BMI, biochemical/metabolic parameters (e.g., cholesterol, glucose, creatinine), and healthcare costs.

**Results:**

547 participants completed the study (Intervention: *n* = 273; Control: *n* = 274). The intervention group achieved a greater reduction in SBP at 12 months (adjusted between-group difference: −3.14 mmHg, 95% CI: −5.24 to −1.03, *P* = 0.004). Subgroup analysis revealed significant heterogeneity by baseline SBP (interaction *P* < 0.001). For participants with baseline SBP below the median (<146 mmHg), the intervention group achieved a significantly larger SBP reduction (between-group difference: −6.79 mmHg, 95% CI: −9.62 to −3.96). It is expected that a decrease of 5 mmHg can reduce the risk of cardiovascular events by about 10%.

## 1 Introduction

### 1.1 Disease burden and management challenges of hypertension

Hypertension is a major risk factor for cardiovascular disease and premature death worldwide, affecting nearly one third of the world's adult population (1). In China, the prevalence of hypertension remains high, but the treatment and control rate is still not ideal, especially in rural areas and resource limited areas (2, 3). The traditional hypertension management model mainly relies on regular outpatient follow-up and patient self-management. In practice, it faces many challenges, such as uneven distribution of medical resources, low patient compliance, poor data continuity and lack of personalized intervention.

### 1.2 Potential and limitations of digital health technology

Digital health technology (DHT), including mobile health (mHealth), wearable devices, remote monitoring and artificial intelligence (AI), provides a new path for the innovation of chronic disease management (4). And digital interventions could be an approach to improve patients' ability to find, evaluate, and use health information (5). DHT can realize continuous monitoring of blood pressure, remote feedback and personalized health education, which is expected to make up for the shortcomings of the traditional model (6). Studies have shown that DHT based interventions, such as remote blood pressure monitoring, can effectively improve short-term blood pressure control (7, 8).

However, most of the existing studies focus on the short-term effect evaluation of a single technical component (such as app or remote monitoring equipment used independently) (7, 9). There is still a lack of sufficient evidence from rigorously designed randomized controlled trials on the long-term effectiveness of integrated DHT platforms that combine features such as remote monitoring, personalized feedback, and patient engagement tools for hypertension management.

### 1.3 Clarify the knowledge gap and research purpose

Therefore, this study aims to fill this knowledge gap. We developed an integrated chronic disease management platform based on DHT, combined with intelligent hypertension management path. Through a randomized controlled trial, we aimed to evaluate the effectiveness of the integrated intervention model in controlling systolic blood pressure in patients with hypertension over a period of 12 months compared with the traditional offline management model. The results of this study will provide a high-level evidence-based basis for the large-scale and integrated application of DHT in the field of chronic disease management.

## 2 Methods

### 2.1 Study design

This study assessed the improvement of hypertension through the Nantong University Affiliated Hospital chronic disease management platform combined with an intelligent hypertension management model. It was a non blind clinical randomized controlled trial conducted among hypertensive adults undergoing physical examinations at the hospital's Health Management Examination Center. The institutional review committees of Nantong University Affiliated Hospital approved the study, and participants provided written informed consent. This study follows the Consolidated Standards of Reporting Trials Extension (CONSORT Extension) reporting guideline.

Recruitment occurred between July 2023, and July 2024. Data collection was completed from Augest 2024 to March 2025. Participants were randomly allocated to the intervention group (intelligent hypertension management mode) or the control group (traditional hypertension management mode). The study population was selected from individuals undergoing physical examinations at the center around July 2023, using their examination data as baseline. The primary outcome measure was SBP at 12 months, adjusted for baseline SBP, gender, and age.

### 2.2 Setting and participants

**Figure 1:**
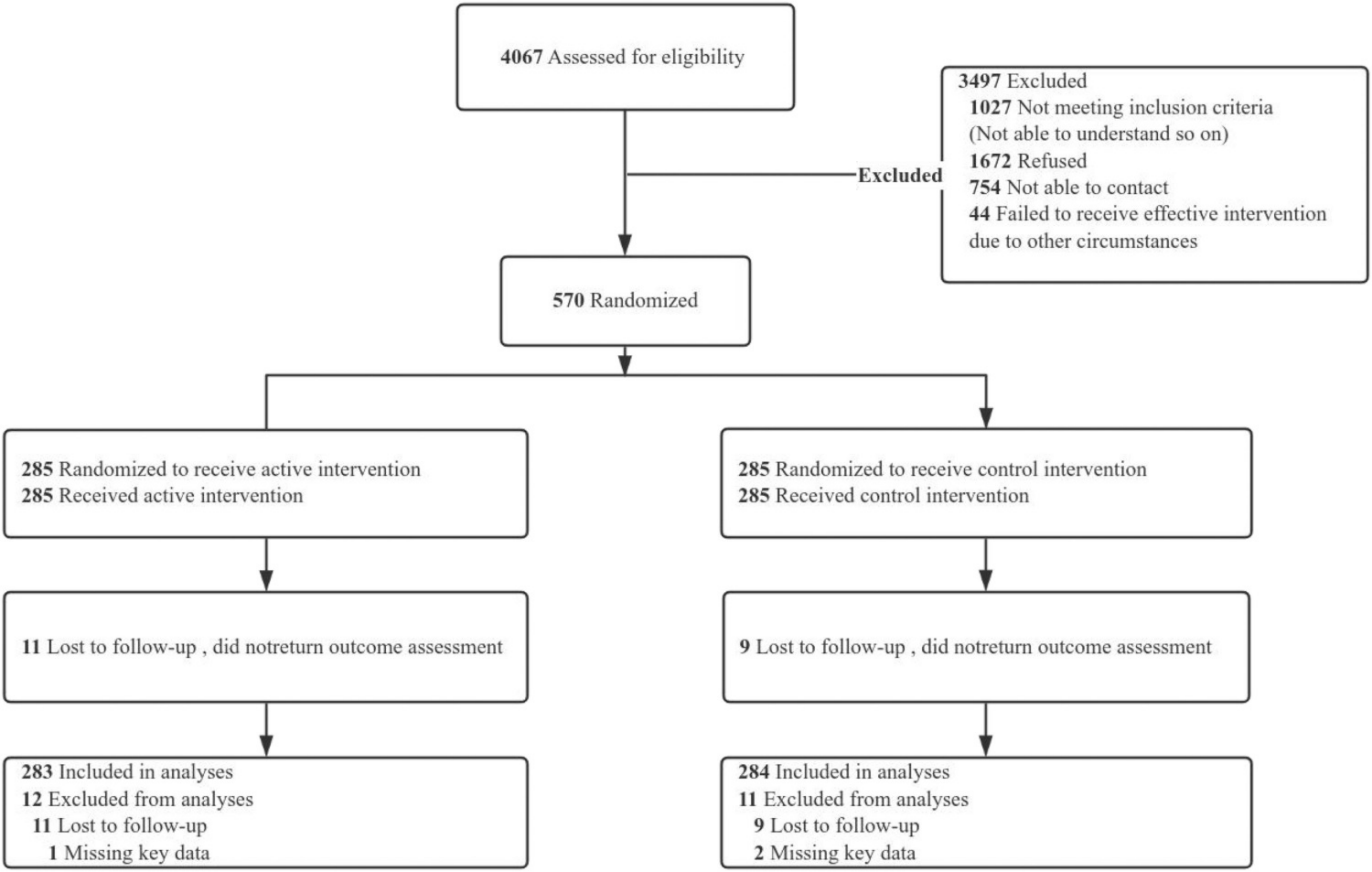
Recruitment, randomization, and participant flow diagram.

Participants were recruited from the physical examination population at the Health Examination Management Center of Nantong University Affiliated Hospital. Eligible participants were adults aged ≥18 years. Exclusion criteria included: secondary hypertension; severe cardiac, cerebral, or renal complications; diagnosed dementia or cognitive impairment; and any other serious comorbidity judged by the physician to preclude effective participation in the study intervention. The CONSORT flowchart (Figure 1) has detailed the recruitment process. Considering the potential white-coat effect and the limitations of single blood pressure measurements during physical examinations, it should be noted that the diagnosis of hypertension is based on the blood pressure values (≥140/90 mmHg) recorded in the EHR and measured by a specialist at least twice during different outpatient visits, or the clinical diagnosis made by the patient currently taking antihypertensive medication, to ensure the accuracy of the diagnosis.

### 2.3 Recruitment and randomization

We screened the EHR data of the 2023 Health Examination Management Center. Based on examination time and research criteria (i.e., patients diagnosed with hypertension by physicians based on multiple elevated blood pressure readings exceeding specific thresholds), individuals meeting these stricter criteria were identified as likely eligible. Randomization was performed after obtaining informed consent. A computer-generated random sequence was used to allocate participants in a 1:1 ratio. Allocation concealment was ensured using sequentially numbered, opaque, sealed envelopes. Due to the behavioral nature of the intervention, blinding of participants and physicians was not feasible. However, outcome assessors were blinded to group assignment throughout the study.

### 2.4 Interventions

#### 2.4.1 Hypertension smart management model (continued management: online DHT-based chronic disease management platform & offline health management and physical examination center)

### 2.5 Hypertension smart management model

**Figure 2:**
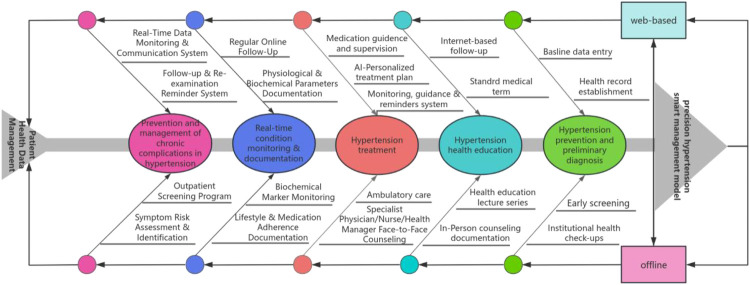
Framework of the intelligent management mode for hypertension.

The intervention group utilized an integrated digital health platform specifically designed for hypertension management at Nantong University Affiliated Hospital. This platform combined online and offline components to deliver a comprehensive, intelligent management model throughout the full lifecycle of hypertension care (10) (Figure 2).

The online component comprised a patient-facing mobile application and a physician management backend, integrated with Internet of Things (IoT) devices (e.g., home smart blood pressure monitors) and AI. AI models facilitate active monitoring of hypertensive patients' health, assisting physicians in adjusting medication plans, treatment strategies, and prevention plans in real-time when necessary (11, 12). This system enabled automatic data upload, real-time monitoring, automated alerts for abnormal values, and personalized health education delivery Health education and follow-up reminders were delivered through multiple synchronized digital channels, including SMS, the mobile app, a WeChat official account, and telephone call (6, 13). Education content was structured and delivered according to a predefined schedule (Table 1) and employed the Teach-Back method to verify patient understanding and provide tailored reinforcement (14, 15). This approach facilitated continuous, interactive patient engagement and self-management support outside the hospital setting, providing feedback on their understanding of hypertension knowledge, related questions, and actual situations regarding diet, exercise, and medication (16).

**Table 1:** Schedule of follow-up education for hypertensive patients.

Merge schedule (educatio*n* + follow-up)		
Time (number of days after receiving the case)	Type	Content
Day 1	Propaganda and education	What is blood pressure?
Day 3	Propaganda and education	Do you know what high blood pressure is?
Day 5	Propaganda and education	Diagnosis of hypertension
Day 7	Follow-up	Persist in measuring blood pressure at home and recording data for doctors to adjust medication
Day 8	Propaganda and education	What is the classification of blood pressure levels?
Day 10	Follow-up	Remind understanding of blood pressure grading and control objectives
Day 11	Propaganda and education	What are the common types of hypertension?
Day 13	Propaganda and education	Special situations where hypertension is easily overlooked
Day 16	Propaganda and education	What are the symptoms of hypertension?
Day 113	Propaganda and education	Common misconceptions about hypertension
Day 114	Propaganda and education	Common Misconceptions of Hypertension 2
Day 115	Propaganda and education	Common Misconceptions of Hypertension 3
Day 116	Propaganda and education	Common Misconceptions of Hypertension 4
Day 118	Propaganda and education	Five common misconceptions about hypertension
On the 120th day	Propaganda and education	Common Misconceptions of Hypertension 6
Day 123	Propaganda and education	Common Misconceptions of Hypertension Seven
Day 125	Follow-up	Remind science to avoid misconceptions about hypertension
On the 180th day	Follow-up	Reminder for Regular Follow up (Nantong University Affiliated Hospital)
The 365th day	Follow-up	Reminder for Annual Review (Nantong University Affiliated Hospital)

The offline component was anchored at the hospital's Health Management and Physical Examination Center. It included in-person health education lectures, face-to-face consultations with specialists, nurses, and health managers, and regular physical examinations for screening and monitoring. Data from these offline encounters (e.g., biochemical test results, medication adjustments) were systematically recorded in the platform.

**Figure 3:**
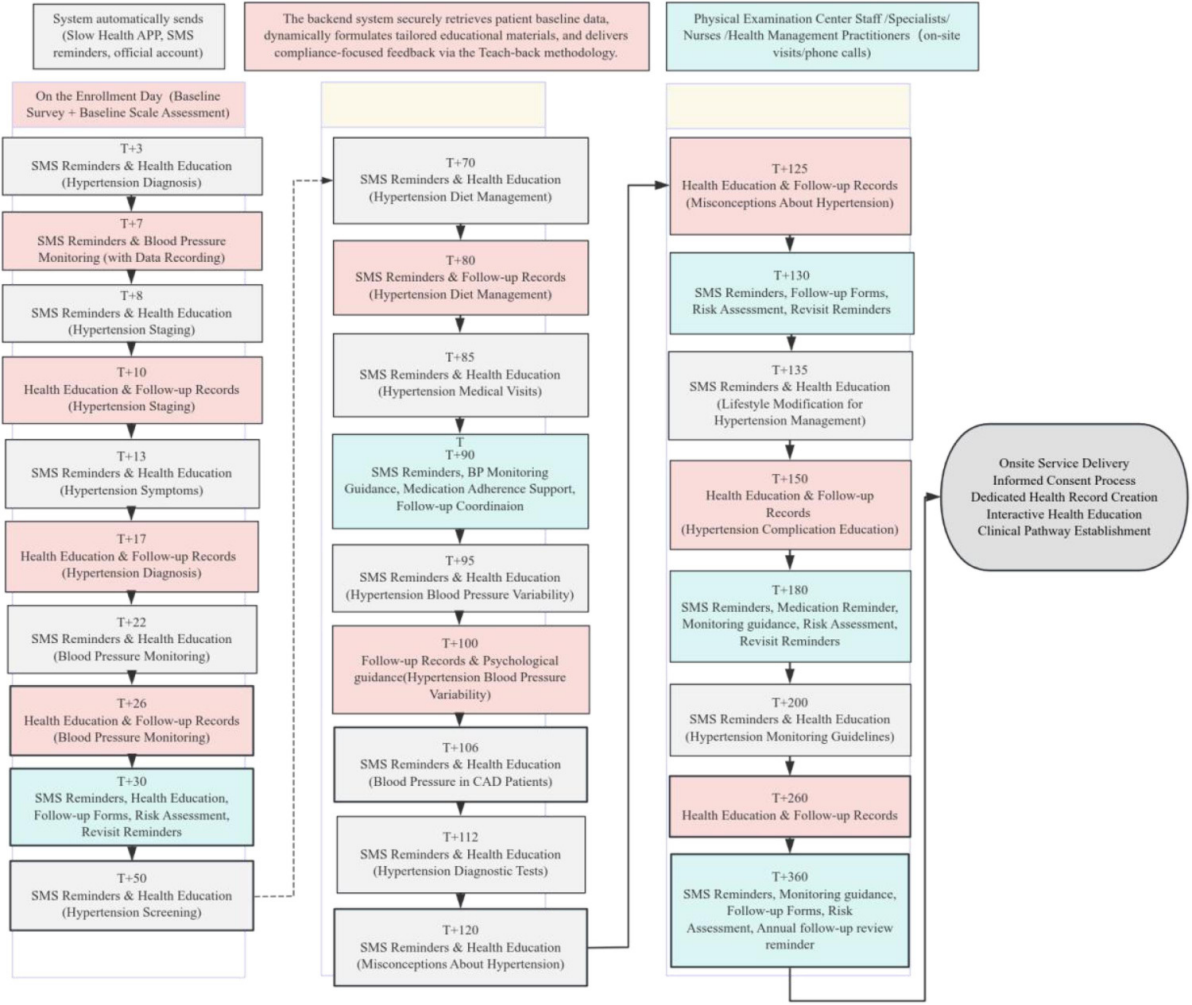
Follow-up pathway diagram for intelligent hypertension management.

The intelligent model operated via a closed-loop, “online monitoring & warning—offline response” system. Data from home-based IoT devices were synchronized in real-time to the platform. Upon triggering predefined warning rules, offline healthcare providers were alerted and could initiate timely interventions, such as phone consultations or scheduling urgent outpatient visits. A structured 360-day follow-up pathway (Figure 3) guided the frequency and content of interactions, including blood pressure measurement reminders, medication adherence prompts, psychological support, and risk assessments.

(Note: A detailed, stage-by-stage description of the intelligent management model, covering Prevention/Preliminary Diagnosis, Health Education, Treatment, Monitoring, Complication Management, and Data Management, is provided in Supplementary Material 1.)

### 2.6 Intelligent management and follow-up mode for hypertension patients

Establishing this mode is key to achieving comprehensive, full-cycle hypertension management. Patients enrolled in the innovative intelligent management platform receive on-site service lectures, sign informed consent, have specialized files created, receive on-site health education, and have management pathways established by professionals (T = enrollment day, T + 3 = 3 days post-enrollment, etc.). Using a 360-day cycle, SMS reminders for blood pressure measurement, health education, follow-up records, medication reminders, psychological guidance, risk assessment, and follow-up scheduling are provided according to the pathway schedule. During the platform intervention period, pre education information should be sent no less than 3 times a week, with a planned follow-up frequency of 11 times. The frequency of related mental health education, follow-up reminders, and other reminders should also be adjusted. Online monitoring data (such as home blood pressure) will be synchronized in real time to the platform. After triggering warnings, offline doctors can intervene in a timely manner through phone calls or the next outpatient visit, forming a closed-loop management of “online monitoring warning offline response” (Figure 3).

#### 2.6.1 Traditional management mode of hypertension (offline management of health management and physical examination center)

The control group received conventional offline hypertension management, including regular outpatient follow-ups, health education, and other related measures. This management covered the following five aspects but with no mandatory requirements, and did not involve any digital platforms or proactive remote follow-ups. These sections are: hypertension prevention and screening (early screening, institutional physical examination), hypertension health education (health education lectures, face-to-face guidance records), hypertension treatment module (outpatient treatment/guidance by specialists/nurses/health managers), real-time monitoring and recording module (biochemical index collection, medication compliance records), and hypertension chronic complication prevention and control module (outpatient screening, symptom risk assessment and identification).

### 2.7 Measurement and outcomes

To avoid self-reporting bias, the evaluation was based on physiological indicators. Primary and secondary outcomes were pre-specified in the study protocol. The primary outcome was systolic blood pressure (SBP) at 12 months. Pre-specified secondary outcomes included: diastolic blood pressure (DBP), body mass index (BMI), a range of biochemical and metabolic parameters (hemoglobin, urea, creatinine, uric acid, total cholesterol, triglycerides, HDL-C, LDL-C, homocysteine, fasting plasma glucose) and total medical examination costs. Data integrity and privacy were ensured by retrieving records from the Health Management and Physical Examination Center's electronic health record data platform.

### 2.8 Sample size and power

The primary outcome was SBP at one cycle (12 months), adjusted for baseline SBP, gender, BMI and age. Sample size calculation used a two-independent-samples *t*-test, with *α* = 0.05 (two-sided) and 90% power. With a two-sided α of 0.05% and 90% power (*β* = 0.1), a standard deviation (*σ*) of 15 mmHg for SBP change (based on prior trials such as the SPRINT study and the ACCORD study), and a target detectable difference (d) of 5 mmHg, the required sample size was 189 participants per group. Accounting for an estimated 20% dropout rate, the planned sample size was 474 (237 per group). To enhance precision given resource availability, we enrolled 570 participants (285 per group).

### 2.9 Statistical analysis

Statistical analyses were performed using STATA 17.0. Quantitative data are presented as mean (standard deviation), and between-group comparisons were conducted using the t-test or Mann–Whitney *U* test based on data distribution; categorical data were analyzed using the *χ*² test. The primary outcome was the change in systolic blood pressure (SBP) at 12 months relative to baseline. Linear regression models were used to compare between-group differences, with adjustment for baseline age, gender, body mass index (BMI), and SBP. Similar analytical methods were applied for continuous secondary outcomes. For baseline comparisons between groups, if the assumption of equal variances was violated (as indicated by Levene's test), Welch's *t*-test was applied. In regression models analyzing outcomes, robust standard errors were used to reduce potential bias from heteroscedasticity.

For missing data due to loss to follow-up, 10 datasets were generated using multiple imputation by chained equations (MICE) in accordance with the intention-to-treat (ITT) principle. Based on the imputed data, generalized estimating equations (GEE) were fitted with an exchangeable working correlation matrix. The interaction term “intervention×time” was tested to evaluate between-group differences in SBP changes from baseline to 12 months, with adjustment for baseline age, gender, SBP, and BMI. Parameter estimates were pooled using Rubin's rules.

The Benjamini-Hochberg method was used to control the false discovery rate for multiple comparisons. Heterogeneity of intervention effects across different subgroups was assessed by introducing interaction terms between the group variable and subgroup variables [including baseline SBP, age, gender, homocysteine (Hcy), and BMI].

### 2.10 Sensitivity analysis

To assess the robustness of the main findings, we performed sensitivity analyses from the following three perspectives:

1. Definition of the analysis dataset: We compared results based on the intention-to-treat (ITT) principle with those from the per-protocol (PP) population. For the PP set, the primary outcome was reanalyzed using the same generalized estimating equations (GEE) model as in the primary analysis, including group, time, their interaction, and adjustment for baseline covariates. The estimated intervention effects (with 95% confidence intervals) from the PP analysis were directly compared with the ITT results.
2. Choice of the core statistical model: We compared estimates from the GEE model with those from a linear mixed-effects model (LMM). The LMM included the same fixed effects as the GEE—group, time, group-by-time interaction, and covariates (age, sex, baseline systolic blood pressure, and baseline BMI).
3. Influence of covariate adjustment strategies: Under the GEE framework, we applied three sequential adjustment levels: Model 1 included only group, time, and their interaction; Model 2 added age and sex; Model 3 further adjusted for baseline systolic blood pressure and baseline BMI. Changes in the estimated intervention effect (interaction coefficient) across models were recorded. Small fluctuations in effect estimates with consistent statistical conclusions would indicate robustness to covariate selection.

## 3 Results

### 3.1 Participants

We identified 4,067 hypertension cases from the EHRs of 89,752 patients examined at the Nantong University Affiliated Hospital Health Management and Physical Examination Center in 2023. Based on on-site physical examination education and sufficient patient communication, Figure 1 illustrates participant recruitment, enrollment, and withdrawal. 570 eligible subjects participated and were randomly assigned to the intervention (*n* = 285) and control (*n* = 285) groups. In the intervention group, 11 were lost to follow-up and 1 had missing data. In the control group, 9 were lost to follow-up and 2 had missing data. Finally, 273 participants were included in the intervention group and 274 in the control group.

### 3.2 Baseline characteristics

A total of 547 participants completed the study: 273 in the intervention group (mean age 65.26 [11.80] years, 171 males [62.64%], 92 [33.70%] with 18.5 ≤ BMI<24, 123 [45.05%] with 24 ≤ BMI<28, 58 [21.25%] with BMI ≥28) and 274 in the control group (mean age 66.19 [10.97] years, 163 males [59.49%], 90 [32.85%] with 18.5 ≤ BMI<24, 117 [42.70%] with 24 ≤ BMI<28, 67 [24.45%] with BMI ≥28). There were no statistically significant differences in age, gender, or BMI between groups (Age: t = 0.95 *P* = 0.341; Gender: *χ*² = 0.57, *P* = 0.450; BMI: *χ*² = 0.818, *P* = 0.664). Baseline SBP was 146.57 (8.15) mm Hg and DBP was 84.77 (6.88) mm Hg in the intervention group, compared to 144.85 (17.11) mm Hg and 83.47 (10.45) mm Hg in the control group, with no statistically significant differences (SBP: *t* = 1.50, *P* = 0.135; DBP: *t* = 1.72, *P* = 0.086) (Table 2).

**Table 2:** Baseline characteristic.

Characteristic	Participants, no. (%)		*t/χ^2^*	*P*
	Intervention (*n* = 273)	Control (*n* = 274)		
Age, mean (SD), y	65.26 (11.80)	66.19 (10.97)	*t* = −0.95	0.341
Gender			*χ^2^* = 0.57	0.450
Male	171 (62.64)	163 (59.49)		
Female	102 (37.36)	111 (40.51)		
BMI			*χ^2^* = 0.818	0.664
18.5 ≤ BMI<24	92 (33.70)	90 (32.85)		
24 ≤ BMI<28	123 (45.05)	117 (42.70)		
28 ≤ BMI	58 (21.25)	67 (24.45)		
Systolic blood pressure, mean (SD), mm Hg	146.57 (8.15)	144.85 (17.11)	*t* *=* 1.50	0.135
Diastolic blood pressure, mean (SD), mm Hg	84.77 (6.88)	83.47 (10.45)	*t* = 1.72	0.086

### 3.3 Changes in blood pressure

Table 3 shows blood pressure changes over 12 months. The primary analysis compared the change in SBP from baseline to 12 months between groups, with adjustment for baseline SBP, age, gender, and body mass index (BMI). In the intervention group, baseline SBP was 146.57 (8.15) mm Hg, and 12-month SBP was 141.01 (12.47) mm Hg [mean change −5.56 (10.56) mm Hg]. In the control group, baseline SBP was 144.85 (17.11) mm Hg, and 12-month SBP was 142.38 (16.61) mm Hg [mean change −2.47 (14.44) mm Hg]. The unadjusted between-group difference in SBP change was −3.09 mm Hg (SE = 1.08, *P* = 0.005); the adjusted difference was −3.14 mm Hg (95% CI: −5.24 to −1.03, *P* = 0.004). The unadjusted between-group difference in DBP change was −1.75 mm Hg (SE = 0.70, *P* = 0.013); the adjusted difference was −1.75 mm Hg (95% CI: −3.12 to −0.38, *P* = 0.012). At 12 months, the proportion of patients achieving SBP<140 mm Hg and DBP<90 mm Hg did not differ significantly between groups (Intervention: 241, 88.28%; Control: 230, 83.94%; OR = 1.44, 95% CI: 0.86–2.43, *χ*² = 2.15, *P* = 0.143) (Table 3).

**Table 3:** Changes in blood pressure.

Variable	Intervention (*n* = 273)			Control (*n* = 274)			Difference, Mean (SE)	*t/χ^2^*	*P*	Adjust Difference Mean (Robust SE) (95%CI)	Adjust *P*
	Baseline	Month12	Change	Baseline	Month12	Change					
Systolic blood pressure, mean (SD), mm Hg	146.57 (8.15)	141.01 (12.47)	−5.56 (10.56)	144.85 (17.11)	142.38 (16.61)	−2.47 (14.44)	−3.09 (1.08)	*t* *=* −2.86	0.005	−3.14 (−1.08) (−5.24 to −1.03)	0.004
Diastolic blood pressure, mean (SD), mm Hg	84.77 (6.88)	81.21 (8.26)	−3.56 (6.37)	83.47 (10.45)	81.65 (10.71)	−1.82 (9.71)	−1.75 (0.70)	*t* = −2.49	0.013	−1.75 (−0.70) (−3.12 to −0.38)	0.012
Blood pressure <140/90 mm Hg, participants, No. (%)	217 (79.49)	241 (88.28)	24 (8.79)	210 (76.64)	230 (83.94)	20 (7.30)	1.44 (0.86 to 2.43)	*χ^2^* = 2.15	0.143	–	–

### 3.4 Subgroup analysis

Given the current lack of *a priori* hypotheses regarding the effects of digital health interventions on patients with different baseline blood pressure levels, we conducted an exploratory subgroup analysis. To obtain approximately equal sample sizes for facilitating comparisons, we used the sample median (146 mmHg) as the cutoff value. These analyses are *post-hoc* and exploratory, and their results should be regarded as hypothesis-generating rather than conclusion-drawing.

Subgroup analysis results indicated a significant interaction effect for baseline SBP groups (*P* < 0.001, significant after FDR correction), indicating heterogeneity in intervention effects across baseline SBP strata (Supplementary Table 4). Baseline SBP < median (<146 mm Hg): The intervention group had a significantly greater SBP reduction at 12 months than the control group (between-group difference −6.79 mm Hg, 95% CI: −9.73 to −3.85). Baseline SBP ≥ median (≥146 mm Hg): No statistically significant difference was observed between groups (between-group difference 1.29 mm Hg, 95% CI: −1.58 to 4.15). The interaction effects for age, gender, H-type hypertension status, and BMI were not significant (*P* > 0.05 after FDR correction), indicating no difference in intervention effect across these subgroups (17) (Table 4).

**Table 4:** Subgroup analysis.

Subgroup	Intervention (*n* = 273)			Control (*n* = 274)			Difference, Mean (95%CI)	*P* value for interaction
	Participants, No	Baseline SBP, mm Hg, Mean (SD)	Month12 SBP, mm Hg, Mean (SD)	Participants, No	Baseline SBP, mm Hg, Mean (SD)	Month12 SBP, mm Hg, Mean (SD)		
SBP								<0.001
At or above median (≥146 mm Hg)	150	152.39 (4.95)	146.04 (12.09)	130	159.52 (10.50)	151.88 (14.66)	1.29 (−1.58 to 4.15)	
Below median (146 mm Hg)	123	139.47 (5.09)	134.87 (9.95)	144	131.62 (9.35)	133.81 (13.29)	−6.79 (−9.73 to −3.85)	
Age								0.849
<60 y	84	141.77 (8.00)	134.86 (10.68)	89	138.61 (14.72)	135.11 (15.22)	−3.42 (−7.20 to 0.36)	
≥60 y	189	148.70 (7.29)	143.74 (12.25)	185	147.86 (17.37)	145.88 (16.15)	−2.98 (−5.55 to −0.41)	
Gender								0.945
Male	171	145.81 (8.28)	140.02 (12.45)	163	143.69 (17.23)	141.04 (17.16)	−3.13 (−5.86 to −0.41)	
Female	102	147.84 (7.82)	142.66 (12.39)	111	146.56 (16.86)	144.35 (15.63)	−2.98 (−6.39 to 0.44)	
Homocysteine								0.691
H-type hypertension, Hcy≥10 umol/L	186	147.13 (8.07)	141.47 (12.78)	234	145.14 (17.13)	142.46 (16.55)	−2.99 (−5.43 to −0.54)	
Not H-type hypertension, Hcy <10 umol/L	87	145.37 (8.26)	140.00 (11.79)	40	143.20 (17.15)	141.93 (17.17)	−4.07 (−8.83 to 0.69)	
BMI								0.170
18.5 ≤ BMI<24	92	146.62 (7.01)	142.45 (10.79)	90	146.44 (16.84)	146.19 (17.04)	−3.92 (−7.59 to −0.25)	
24 ≤ BMI<28	123	146.17 (8.92)	139.01 (13.29)	117	143.45 (17.25)	140.74 (15.58)	−4.23 (−6.64 to −1.81)	
28 ≤ BMI	58	147.34 (8.22)	142.91 (12.73)	67	145.16 (17.27)	140.12 (17.12)	0.61 (−3.82 to 5.05)	

### 3.5 Secondary outcomes

Secondary outcomes were adjusted for covariates (age, gender, baseline value, BMI) and FDR correction for multiple comparisons (Benjamini-Hochberg method, FDR = 5%). The between-group differences in 12-month changes for HDL-C (17) and medical costs remained significant after covariate adjustment and FDR correction. Compared to the control group, the intervention group showed: Increased HDL-C by 0.04 mmol/L (adjusted difference 0.04 mmol/L, 95% CI: 0.02–0.07, *P* = 0.003, *q* = 0.017)) Increased medical costs by 222.02 units (adjusted difference 222.02, 95% CI: 121.96–322.07, *P* < 0.001, *q* = 0.001). Regarding the minimal change (0.04 mmol/L) in HDL-C, we have provided a cautious interpretation in the discussion and refrained from overstating its clinical significance. The medical costs in this study mainly refer to the participants' direct examination fees. This is to preliminarily explore whether the participants' acceptance of intervention (for either blood pressure reduction or elevation) affects the subsequent number of examinations and examination costs. All costs are calculated in Renminbi (CNY). After FDR correction (*q* > 0.05), no other secondary outcomes showed statistically significant differences (Table 5).

**Table 5:** Secondary outcome.

Outcome	Mean (SD)				Difference, Mean (SE)	*t*	*P*	Adjust Difference, Mean (95%CI)	Adjust *P*	*q*
	Intervention (*n* = 273)		Control (*n* = 274)							
	Baseline	Month12	Baseline	Month12						
Hemoglobin	146.77 (13.18)	145.65 (13.58)	147.66 (13.51)	145.66 (12.90)	0.88 (0.61)	*t* = 1.43	0.151	0.56 (−0.59 to 1.70)	0.337	0.591
Carbamide	5.73 (1.27)	5.87 (1.45)	5.74 (1.44)	5.80 (1.51)	0.08 (0.11)	*t* = 0.71	0.476	0.08 (−1.12 to 0.27)	0.439	0.603
Creatinine	73.10 (16.35)	73.56 (17.04)	72.58 (15.76)	73.65 (16.04)	−0.60 (0.62)	*t* = −0.97	0.332	−0.54 (−1.73 to 0.65	0.376	0.591
Uric acid	363.23 (82.65)	362.34 (88.95)	374.90 (84.65)	364.26 (81.55)	9.76 (5.57)	*t* = 1.75	0.080	5.31 (−4.81 to 15.43)	0.303	0.591
Total cholesterol	5.35 (1.15)	5.05 (1.09)	5.08 (1.14)	4.90 (1.09)	−0.13 (0.08)	*t* = −1.68	0.094	−0.30 (−0.16 to 0.11)	0.666	0.733
Trilaurin	1.74 (1.11)	1.77 (1.32)	1.84 (1.24)	1.81 (1.19)	0.06 (0.09)	*t* = 0.64	0.519	0.02 (−0.15 to 0.18)	0.823	0.823
HDL-C	1.39 (0.31)	1.40 (0.31)	1.38 (0.31)	1.35 (0.31)	0.04 (0.02)	*t* = 2.74	0.006	0.04 (0.02 to 0.07)	0.003	0.017
LDL-C	3.50 (0.88)	3.25 (0.83)	3.28 (0.87)	3.17 (0.84)	−0.13 (0.06)	*t* = −2.73	0.026	−0.05 (−0.16 to 0.05)	0.309	0.591
FPG	5.98 (1.28)	5.75 (1.11)	5.92 (1.23)	5.68 (1.08)	0.01 (0.08)	*t* = 0.16	0.873	0.05 (−0.09 to 0.18)	0.501	0.612
Hcy	11.78 (4.56)	13.53 (6.36)	13.36 (6.11)	13.78 (4.37)	1.33 (0.38)	*t* = 3.50	<0.001	0.73 (0.05 to 1.41)	0.036	0.132
Cost	2,411.04 (839.52)	2,071.43 (642.84)	2,837.84 (707.46)	1,893.89 (539.43)	604.34 (54.51)	*t* = 7.97	<0.001	222.02 (121.96 to 322.07)	<0.001	0.001

HDL-C, high density lipoprotein cholesterol; LDL-C, low-density lipoprotein cholesterol; FPG, fasting plasma glucose; Hcy, homocysteine.

### 3.6 Sensitivity analysis

The sensitivity analysis results are shown in Table 6. Under different analysis scenarios, the parameter (*β*) representing the net effect of the intervention remained highly stable. Firstly, comparing the results of the intention to treat analysis set (ITT) and the compliance set (PP) analysis, the estimated intervention effects were very close (ITT: *β* = 3.25, 95% CI: 1.13–5.37, *p* = 0.003; PP: *β* = 3.14, 95% CI: 1.03–5.24, *p* = 0.004). Secondly, when using the linear mixed effects model (LMM) instead of the generalized estimation equation (GEE) for analysis, the estimated values of intervention effects remain unchanged (*β* = 3.14) and the confidence intervals are similar (95% CI: 1.12–5.15, *p* = 0.002). Finally, we tested different covariate adjustment strategies. From a model that only included core variables (unadjusted, *β* = 3.14) to a fully adjusted model that gradually adjusted for age, gender, baseline systolic blood pressure, and further adjusted for baseline BMI, there were no substantial changes in the estimated values, accuracy (standard error), and statistical significance of intervention effects (*β* = 3.14, *p* = 0.004 in all models).

**Table 6:** Sensitivity analysis of intervention effects (intervention vs. control).

Scenario	Description	*β*	*SE*	*t*	*P*	95%CI
PP	GEE (PP)	3.14	1.07	2.92	0.004	(1.03–5.24)
ITT	GEE (ITT)	3.25	1.08	3.00	0.003	(1.13–5.37)
GEE vs. LMM	LMM(PP)	3.14	1.03	3.05	0.002	(1.12–5.15)
^a^Model1	GEE (PP)	3.14	1.07	2.92	0.004	(1.03–5.24)
^b^Model2	GEE (PP)	3.14	1.07	2.92	0.004	(1.03–5.24)
^c^Model3	GEE (PP)	3.14	1.07	2.92	0.004	(1.03–5.24)

SE, standard error; CI, confidence interval; PP, per-protocol; ITT, intention-to-treat; GEE, generalized estimating equation; LMM, linear mixed-effects model; SBP, systolic blood pressure.

β represents the adjusted between-group difference in the change in systolic blood pressure from baseline to 12 months, calculated as intervention group minus control group. A negative β indicates a greater reduction in SBP in the intervention group.

a Model unadjusted.

b Model adjusted: age, gender, baseline (SBP).

c Model adjusted: age, gender, baseline (SBP) and BMI.

## 4 Discussion

### 4.1 Principal findings and interpretation

This randomized controlled trial evaluated the efficacy of an intelligent hypertension management mode (intervention group) compared to traditional management (control group) in blood pressure control. The results demonstrated that both management modes led to reductions in blood pressure; however, the intervention group exhibited significantly greater decreases in systolic blood pressure (SBP) and diastolic blood pressure (DBP) after 12 months. A comprehensive DHT-based management platform combined with an intelligent hypertension care model led to a statistically significant reduction in systolic blood pressure (−3.14 mmHg, 95% CI: −5.24 to −1.03) compared to traditional management over 12 months. This finding suggests that integrated digital interventions can provide a small, incremental benefit in blood pressure control. The effect was more pronounced in the exploratory subgroup of participants with baseline SBP below 146 mmHg, indicating that such an approach might be particularly suitable for patients with milder, earlier-stage hypertension, potentially serving as an effective tool for intensive lifestyle intervention and prevention of disease progression.

### 4.2 Comparison with existing literature

Our results are consistent with the growing body of evidence on digital health interventions for hypertension, which often report modest BP reductions (7). Relevant studies have found that digital interventions for health behaviors among hypertensive patients based on intelligent health promotion systems and WeChat play a role in improving specific health outcomes and adherence to health behaviors in elderly hypertensive individuals (18). A recent meta-analysis concluded that tailored initiatives that leverage digital health may have the potential to advance equity in hypertension outcomes (19). The smaller-than-hypothesized effect size in our study (observed 2.3 mmHg vs. assumed 5 mmHg) may be attributed to the intensive comparison received by the control group, which continued to receive standard care within a tertiary hospital setting. This “high standard of usual care” potentially diminishes the apparent marginal benefit of the intervention. Furthermore, while previous studies have frequently focused on short-term efficacy of single-component tools (e.g., remote monitoring only), our study adds value by evaluating a multi-component, integrated platform over a longer 12-month period, providing insights into its sustained, real-world application.

The exploratory subgroup analysis suggesting greater benefit in patients with lower baseline SBP aligns with the concept that digital interventions may be most effective before the establishment of severe, often multifactorial, hypertension. This finding, however, requires confirmation in future studies pre-specifying such subgroups.

### 4.3 Clinical and public health implications

From a clinical perspective a 2–3 mmHg SBP reduction at the individual level may seem limited. However, from a public health and population standpoint, a shift of this magnitude across a large hypertensive population is projected to translate into a meaningful reduction in cardiovascular events (20). The intervention's strength lies in its ability to provide continuous, scalable support and education, potentially improving patient engagement and self-management literacy over the long term (5).

### 4.4 Limitations and future directions

The interpretation of our findings must be considered in the context of several limitations.

First, the assumed effect size for sample calculation was optimistic, and the observed smaller effect indicates that the study may have been underpowered for some secondary outcomes or more subtle effects. Second, due to the behavioral nature of the intervention, blinding of participants and care providers was not feasible, which may have introduced performance bias. Although outcome assessors were blinded, the Hawthorne effect could have influenced participant behavior. Third, we observed a baseline imbalance in SBP distributions and a substantially larger standard deviation in the control group compared to the intervention group. Although statistical models adjusted for baseline values, this heterogeneity in variance may have reduced the precision of our primary effect estimate. Fourth, the study lacked objective measures of key behavioral mediators, such as medication adherence, physical activity, and dietary changes, limiting our ability to explain the exact mechanisms behind the observed BP changes. Fifth, the generalizability of our findings is constrained by the recruitment of a predominantly urban, tech-literate population from a single tertiary hospital center, which may not represent rural populations or those with lower digital literacy. Sixth, there was a potential for contamination, as control group participants might have independently accessed other digital health resources. Finally, the economic implications remain unclear due to the unexplained increase in healthcare costs within the intervention group, necessitating a formal cost-effectiveness analysis.

## 5 Conclusion

In conclusion, this study provides evidence that an integrated DHT-based chronic disease management platform can yield a statistically significant improvement in systolic blood pressure control (mean difference −3.14 mmHg) among hypertensive patients over 12 months, with a signal of greater benefit in those with milder hypertension. While the clinical effect is modest, the scalable nature of digital interventions offers a potential avenue for broadening the reach of hypertension management. Future work should focus on optimizing the intervention's efficiency, demonstrating its cost-effectiveness, and validating its efficacy in broader, more diverse healthcare settings (21).

Future efforts should integrate mobile health, Internet-based platforms, and big data analytics to extend these solutions to primary care settings.

## Data Availability

The raw data supporting the conclusions of this article will be made available by the authors, without undue reservation.
