# Supplementary material for: A randomized controlled trial on the application of a chronic disease management platform based on digital health technology combined with an innovative model of intelligent management for hypertension in patients with hypertension
Source: Front Digit Health. 2026 Jan 26;7:1678235. doi: 10.3389/fdgth.2025.1678235 (PMC12883777; doi:10.3389/fdgth.2025.1678235)
Supplement: Supplementary file 1 [file Supplementaryfile1.docx]

**Translation of Intervention Measures**

Affiliated Hospital of Nantong University has initiated the development of a Health Management Information Platform, on which a brand-new integrated online-offline chronic disease management program is built. This platform enables real-name registration for patients, establishment of comprehensive medical records, patient categorization by disease treatment teams, formulation of follow-up plans, and cohort-specific follow-up management. Specifically developed as a software platform to promote the hospital's health management services, it is currently undergoing a trial among diabetic patients (see Figures 1 and 2) and operating smoothly. Its backend has been connected to the hospital's Laboratory Information System (LIS) and Physical Examination Information System. Further efforts are being made to quantitatively assess the significance of the health management platform, as well as the feasibility and effectiveness of its implementation strategies. Registered patients can access disease prevention and health care knowledge through popular science articles, short videos, health lectures, and other resources.

Figure 1: Platform Login Interface[Image Caption: Personal Whole-Life Cycle Standardized Health Management Service Platform of Affiliated Hospital of Nantong University]


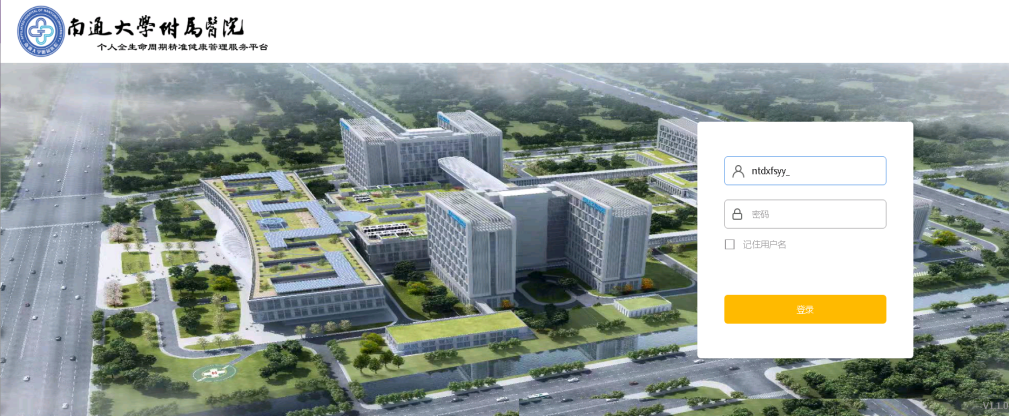


Figure 2: Patient Management Interface within the Platform


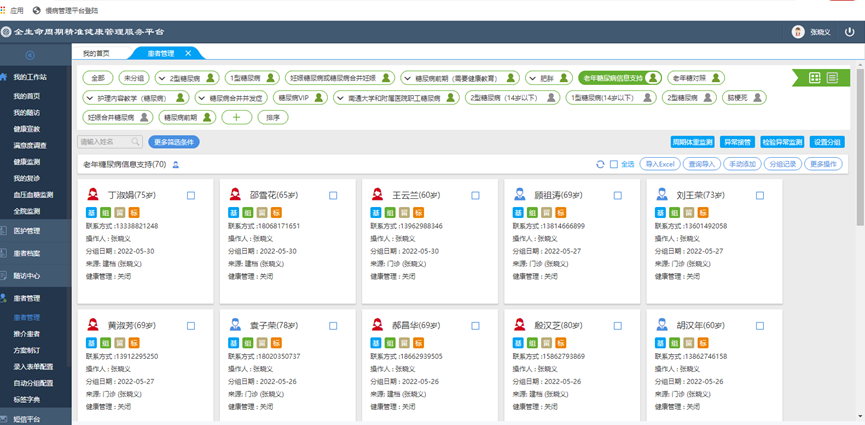


**Intervention Measures**

Smart Management Model for Hypertension (Continuous Management Integrating Online Management via Digital Health Technology-Based Chronic Disease Management Platform and Offline Management by Health Management and Physical Examination Center)

Chronic Disease Management Platform (Hypertension) of Affiliated Hospital of Nantong University: The path design for the innovative smart hypertension management model. The smart medical and health management platform comprises four layers: the Infrastructure Layer, Support Layer, Application Layer, and Access Layer.

Infrastructure Layer: Includes various hardware facilities such as application servers, database servers, and basic networks.

Support Layer: Encompasses AI basic capability engines, AI scenario implementation services, AI business functions, AI capability open platforms, and knowledge base management.

Application Layer: Features a business foundation platform, supporting applications, etc., enabling system management, patient record management, chronic disease group management, health education, online consultation, blood pressure monitoring, and more.

Access Layer: Consists of the hypertension management platform, APPs for medical staff and patients, and WeChat service platforms.

Overall Architecture of Hypertension Management on the Chronic Disease Management Platform: Employs "Internet + Internet of Things (IoT) + Artificial Intelligence (AI)" to achieve intelligent, personalized, and refined hypertension management.

In-Hospital Management

The smart medical platform for hypertension health management can be connected to the existing information systems of medical institutions, enabling continuous in-hospital tracking and timely intervention for hypertension patients. Doctors can utilize an intelligent health assessment system that integrates physical examination data, monitoring data, lifestyle information, and medical history to assist in the diagnosis of related diseases. Additionally, AI models are used to conduct proactive health monitoring of hypertension patients, and when necessary, assist doctors in real-time adjustments to medication plans, treatment plans, and prevention programs.

Out-of-Hospital Management

Through the Hypertension Health Management Platform, follow-up health care and intervention plans outside the hospital are implemented. In-hospital doctors can leverage IoT devices such as smart bracelets, smart blood pressure monitors, blood glucose meters, and integrated health management devices in patients' homes. Combined with mobile APPs, WeChat official accounts, SMS, and other channels, and based on the data that hypertension patients manually synchronize to the chronic disease management platform, personalized health guidance is provided to patients. This enables real-time monitoring reminders, remote health guidance, and home care guidance for patients.

**Design of the Innovative Smart Hypertension Management Model**

Comprehensive health management for hypertension covers the prevention, screening, assessment, diagnosis, education, monitoring, and treatment of hypertension, as well as the prevention, diagnosis, monitoring, and treatment of chronic complications of hypertension. It adopts an integrated online-offline approach to achieve whole-life-cycle management for hypertension patients (see Figure 3).

Hypertension Prevention and Preliminary Diagnosis Stage: This stage primarily targets patients with early-stage hypertension or high-risk individuals, aiming for early screening to achieve primary prevention. It adopts a model of online diagnosis and prevention combined with offline screening and assessment. Online activities focus on baseline data entry, health record establishment, and supplementary risk identification. Offline efforts center on early screening and physical examinations at the hospital's Physical Examination Center, with symptom assessment as a supplement. These measures enhance public participation and awareness in hypertension prevention and control. By implementing early intervention, preventing risk factors, and conducting health management, the focus of hypertension prevention and control is shifted from disease treatment to pre-onset prevention, thereby reducing the incidence of hypertension and the risk of complications.

Hypertension Health Education Stage (see Table 1): During this stage of smart hypertension management, real-time doctor-patient interaction is realized through the digital health technology-based chronic disease management platform. The core of hypertension health education lies in "early prevention, early detection, and early treatment"—improving the public's awareness of hypertension and patients' self-management capabilities, advocating healthy lifestyles, promoting early prevention and standardized management, and reducing cardiovascular and cerebrovascular risks. Online, the system disseminates health knowledge via multiple channels including SMS, mobile APPs, WeChat official accounts, and phone calls. Meanwhile, real-time feedback and online follow-ups are conducted based on the Teach-back method. Offline, health education lectures are held, where medical staff provide face-to-face instruction on hypertension-related knowledge and prevention measures. The educational content includes:

General knowledge about hypertension: concept, clinical symptoms, types, blood pressure classification and control targets, correct blood pressure measurement methods, hazards of hypertension and prevention of complications, importance of self-management for hypertension, rational diet, smoking cessation and alcohol restriction, appropriate exercise for hypertension patients, blood pressure fluctuations, hypertension examination items, and common misunderstandings about hypertension.

Personalized educational intervention plans: personalized target achievement rates for relevant indicators, personalized lifestyle and dietary interventions, and personalized medication prescriptions.

Based on the aforementioned Teach-back health education model, patients can communicate and provide feedback to medical staff in real time (both online and offline) regarding their learning progress of hypertension knowledge, related questions, and actual conditions in terms of diet, exercise, and medication. This provides a basis for professionals and the chronic disease management platform to further develop personalized diagnosis and treatment plans, thereby achieving a long-term positive cycle.

Hypertension Treatment Stage: Offline treatment mainly takes place in outpatient clinics, with consultations conducted by specialists, nurses, and health managers to ensure timely treatment. Online, based on platform monitoring data—such as changes in home-measured blood pressure and patient weight—personalized intervention plans are adjusted in real time. The platform also accurately records patients' medication usage, provides medication guidance, and facilitates further efficacy observation.

Real-Time Condition Monitoring and Recording Stage: The offline Health Management Center conducts regular physical examination screenings and collects relevant biochemical indicators. These data are entered into the online chronic disease platform, and online-offline follow-ups are carried out to record medication adherence and relevant physiological indicators. This ensures the connection of online-offline information and doctor-patient interaction.

Prevention and Control Stage for Chronic Complications of Hypertension: Regular screenings for chronic complications of hypertension are provided based on patients' blood pressure management status. The online backend captures data for real-time monitoring and communication, and sends reminders for re-examinations. Offline, outpatient screenings and symptom risk assessments are conducted.

Patient Personal Data Management Stage: All data collected from hypertension patients throughout their participation in the whole-cycle health management (both online and offline) are gathered and recorded. Online data include home-measured blood pressure and blood glucose levels, weight, follow-up records, and assessment data obtained from questionnaires sent by the platform. Offline data include relevant biochemical indicators from hypertension assessments during outpatient re-examinations.

A diagrammatic representation of the framework for intelligent hypertension management models：[Image Caption:Online: Regular Online Follow-ups; Network Follow-ups; Backend Data Capture for Real-Time Monitoring and Communication; Medication Guidance and Supervision; Baseline Data Entry; System-Sent Health Knowledge; Health Record Establishment; Re-Examination Reminders; AI Personalized Plans; Refined Smart Hypertension Management Model; Monitoring, Guidance, and RemindersHypertension: Preliminary Diagnosis; Health Education; Treatment; Real-Time Condition Monitoring and Recording; Chronic Complication PreventionOffline: Outpatient Screenings; Outpatient Treatment; Early Screening and Identification; Biochemical Indicator Collection; Health Education Lectures; Symptom Risk Assessment; Lifestyle and Medication Adherence Recording; Institutional Physical Examinations; Face-to-Face Guidance by Specialists/Nurses/Health Managers; Face-to-Face Guidance Records]

**Smart Follow-Up Model for Hypertension Patients**

The establishment of a smart follow-up model for hypertension patients is crucial and serves as an effective basis for achieving comprehensive, whole-cycle hypertension management. For patients selected to be included in the innovative smart hypertension management platform, professionals conduct on-site service briefings, obtain signed informed consent forms, establish special records, provide on-site health education, and develop management paths (where "T" represents the enrollment day, "T+3" represents the 3rd day after enrollment, and so on). With a 360-day cycle, the platform sends SMS reminders, guides blood pressure measurement, delivers health education, keeps follow-up records, sends medication reminders, provides psychological guidance, conducts risk assessments, and issues re-examination reminders in accordance with the path schedule. This model enables real-time health information exchange between medical staff and patients, realizing continuous integrated online-offline management

Smart Hypertension Management Follow-Up Path Diagram

Enrollment Day (Baseline Survey, Baseline Scale Assessment)

T+3: SMS Reminder, Health Education (Hypertension Diagnosis)

T+8: SMS Reminder, Health Education (Blood Pressure Classification)

T+10: Health Education, Follow-Up Record (Blood Pressure Classification)

T+13: SMS Reminder, Health Education (Hypertension Symptoms)

T+17: Health Education, Follow-Up Record (Hypertension Diagnosis)

T+22: SMS Reminder, Health Education (Blood Pressure Measurement)

T+26: Health Education, Follow-Up Record (Blood Pressure Measurement)

T+30: SMS Reminder, Health Education (Hypertension Examination Items)

T+50: SMS Reminder, Health Education (Hypertension Examinations)

T+70: SMS Reminder, Health Education (Hypertension Diet)

T+80: SMS Reminder, Blood Pressure Measurement (Data Recording)

T+85: SMS Reminder, Follow-Up Form, Risk Assessment, Re-Examination Reminder

T+90: SMS Reminder, Monitoring Guidance, Medication Guidance, Re-Examination Reminder

T+95: SMS Reminder, Health Education (Blood Pressure Fluctuations)

T+100: Follow-Up Record, Psychological Guidance (Blood Pressure Fluctuations)

T+106: SMS Reminder, Health Education (Blood Pressure for Coronary Heart Disease Patients)

T+112: SMS Reminder, Health Education, Follow-Up Form, Risk Assessment, Re-Examination Reminder

T+120: SMS Reminder, Health Education (Hypertension Misconceptions)

T+125: Health Education, Follow-Up Record (Hypertension Misconceptions)

T+130: SMS Reminder, Follow-Up Record (Hypertension Diet)

T+135: SMS Reminder, Health Education (Medical Consultation for Hypertension)

T+150: Health Education, Follow-Up Record (Complication Education)

T+180: SMS Reminder, Medication Reminder, Monitoring Guidance, Risk Assessment, Re-Examination Reminder

T+200: SMS Reminder, Health Education (Monitoring Guidance)

T+260: SMS Reminder, Health Education (Lifestyle Education)

T+360: SMS Reminder, Monitoring Guidance, Follow-Up Form, Risk Assessment, Annual Re-Examination Reminder

On-Site Service Briefing, Informed Consent Signing, Special Record Establishment, On-Site Health Education, Management Path Development]

**Traditional Hypertension Management Model (Offline Management by Health Management and Physical Examination Center)**

The traditional hypertension management model consists of five modules. Unlike the aforementioned intervention model, it does not rely on the assistance of a chronic disease management platform and mainly relies on offline management by the Health Management and Physical Examination Center of Nantong University. The five modules are as follows:

Hypertension Prevention and Screening Module: Early screening and institutional physical examinations.

Hypertension Health Education Module: Health education lectures and face-to-face guidance records.

Hypertension Treatment Module: Outpatient treatment and face-to-face guidance by specialists, nurses, and health managers.

Real-Time Condition Monitoring and Recording Module: Biochemical indicator collection and medication adherence recording.

Prevention and Control Module for Chronic Complications of Hypertension: Outpatient screenings, symptom risk assessment, and identification.

**Detailed follow-up education schedule**

Comprehensive Integrated Schedule (Health Education + Follow-Up)

Time (Days After Enrollment) Type Content

Day 1 Health Education What is blood pressure?

Day 3 Health Education Do you know what hypertension is?

Day 5 Health Education Diagnosis of hypertension

Day 7 Follow-Up Insist on measuring blood pressure at home and record data for doctors to adjust medications

Day 8 Health Education How is blood pressure classified by level?

Day 10 Follow-Up Reminder to understand blood pressure classification and control targets

Day 11 Health Education What are the common types of hypertension?

Day 13 Health Education Easily overlooked special cases of hypertension

Day 16 Health Education What symptoms indicate hypertension?

Day 17 Follow-Up Reminder to seek timely medical attention for early hypertension diagnosis

Day 19 Health Education Who is at risk of hypertension?

Day 22 Health Education Preparation before blood pressure measurement

Day 25 Health Education Correct posture for blood pressure measurement

Day 26 Follow-Up Reminder to pay attention to preparation before blood pressure measurement

Day 28 Health Education Why hypertension requires attention

Day 30 Follow-Up Reminder for regular re-examination

Day 31 Health Education Office blood pressure treatment targets for different populations

Day 36 Health Education Beware of secondary hypertension

Day 40 Health Education Why do hypertension patients need comprehensive examinations?

Day 44 Health Education Frequency of blood pressure measurement

Day 47 Health Education Hyperlipidemia: the "accomplice" of hypertension

Day 51 Health Education Rational diet

Day 54 Health Education Why is a rational diet necessary?

Day 57 Health Education Why reduce salt intake?

Day 61 Health Education How to control salt intake?

Day 64 Health Education Watch out for "hidden salt" in food

Day 67 Health Education How to choose cooking oil?

Day 70 Health Education Why weight loss is important and its benefits for hypertension

Day 73 Health Education Why quit smoking and how to do it?

Day 76 Health Education Can hypertension patients drink alcohol?

Day 79 Health Education Do hypertension patients need physical exercise?

Day 80 Follow-Up Reminder for rational diet, smoking cessation, alcohol restriction, and appropriate exercise

Day 82 Health Education Dangerous movements that hypertension patients should avoid during exercise

Day 85 Health Education Fruits and vegetables suitable for hypertension patients

Day 88 Health Education Symptoms of hypertension that require timely medical attention

Day 90 Follow-Up Reminder for regular re-examination (Affiliated Hospital of Nantong University)

Day 91 Health Education Does blood pressure fluctuate with seasons?

Day 94 Health Education Does blood pressure fluctuate throughout the day?

Day 97 Health Education What factors cause blood pressure fluctuations?

Day 100 Health Education Why monitor 24-hour ambulatory blood pressure?

Day 100 Follow-Up Reminder not to adjust medications arbitrarily due to weather changes

Day 103 Health Education Risk factors for cardiovascular and cerebrovascular events in hypertension patients

Day 106 Health Education How to manage morning blood pressure in coronary heart disease patients?

Day 109 Health Education What does proteinuria mean for hypertension patients?

Day 112 Health Education Which regular examinations do hypertension patients need?

Day 113 Health Education Common Misconception 1 about Hypertension

Day 114 Health Education Common Misconception 2 about Hypertension

Day 115 Health Education Common Misconception 3 about Hypertension

Day 116 Health Education Common Misconception 4 about Hypertension

Day 118 Health Education Common Misconception 5 about Hypertension

Day 120 Health Education Common Misconception 6 about Hypertension

Day 123 Health Education Common Misconception 7 about Hypertension

Day 125 Follow-Up Reminder to scientifically avoid misunderstandings about hypertension

Day 180 Follow-Up Reminder for regular re-examination (Affiliated Hospital of Nantong University)

Day 365 Follow-Up Reminder for annual re-examination (Affiliated Hospital of Nantong University)

**Diagram of Patient Categorization Management**

The specific plans within the designed patient personal data management module, hypertension prevention and diagnosis module, hypertension health education module, hypertension treatment and condition monitoring module, and hypertension chronic complication prevention and control module are integrated to form a regionalized and grouped health management platform.

**
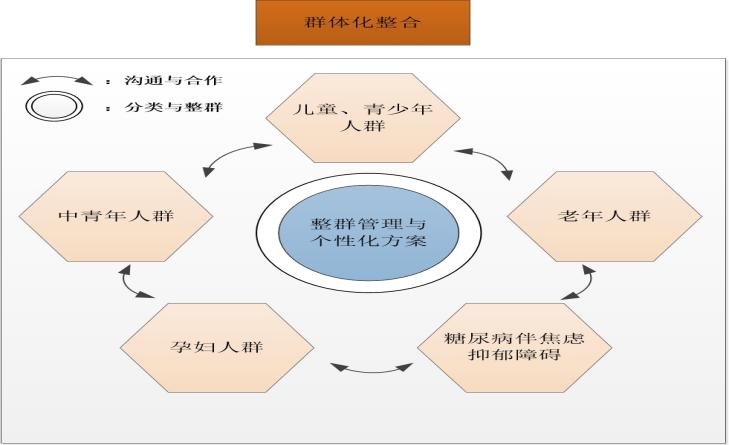
**
