# Supplementary material for: A randomized controlled trial on the application of a chronic disease management platform based on digital health technology combined with an innovative model of intelligent management for hypertension in patients with hypertension
Source: Front Digit Health. 2026 Jan 26;7:1678235. doi: 10.3389/fdgth.2025.1678235 (PMC12883777; doi:10.3389/fdgth.2025.1678235)
Supplement: Supplementary file 2 [file Datasheet1.pdf]

Supplementary Material

Supplementary content:

Table S1. Linear regression of blood pressure changes

Table S2. Benjamin-Hochberg method for subgroup analysis

Table S1. Linear regression of blood pressure changes

Variable	*β*	*95%CI*	*P*
**Systolic blood pressure, SBP**			
Age	0.19	0.10 to 0.28	<0.001
BMI	-0.36	-0.66 to -0.05	0.021
Baseline SBP	0.60	0.52 to 0.68	<0.001
Gender			
Male	1.00		
Female	0.71	-1.31 to 2.73	0.490
Group			
Intervention	1.00		
Control	2.30	0.35 to 4.25	0.021
**Diastolic blood pressure, SBP**			
Age	-0.13	-0.19 to -0.07	<0.001
BMI	-0.15	-0.34 to 0.05	0.142
Baseline DBP	0.58	0.50 to 0.66	<0.001
Gender			
Male	1.00		
Female	-0.92	-2.26 to 0.42	0.179
Group			
Intervention	1.00		
Control	13.94	0.13 to 2.66	0.031

Table S2. Benjamin-Hochberg method for subgroup analysis

Subgroup	*P* value for interaction	FDR_critical	*q*
SBP	<0.001	0.01	0.0005
At or above median (≥146 mm Hg)			
Below median (< 146 mm Hg)			
Age	0.849	0.02	0.9448
< 60 y			
≥60 y			
Gender	0.945	0.03	0.9448
Male			
Female			
Homocysteine	0.691	0.04	0.9448
H-type hypertension, Hcy≥10umol/L			
Not H-type hypertension, Hcy<10umol/L			
BMI	0.170	0.05	0.42375
18.5≤BMI＜24			
24≤BMI＜28			
28≤BMI			

Abbreviation：HDL-C, High density lipoprotein cholesterol; LDL-C, Low-Density Lipoprotein Cholesterol; FPG, Fasting Plasma Glucose; Hcy, Homocysteine.
